# Sexual dimorphism in myocardial acylcarnitine and triglyceride metabolism

**DOI:** 10.1186/s13293-016-0077-7

**Published:** 2016-05-13

**Authors:** Sriram Devanathan, Timothy D. Whitehead, Nicole Fettig, Robert J. Gropler, Samuel Nemanich, Kooresh I. Shoghi

**Affiliations:** Department of Radiology, Washington University in St. Louis, 510 South Kingshighway Blvd., Campus Box 8225, Saint Louis, MO 63110 USA; Department of Medicine, Washington University in St. Louis, 510 South Kingshighway Blvd., Campus Box 8225, Saint Louis, MO 63110 USA; Department of Biomedical Engineering, Washington University in St. Louis, 510 South Kingshighway Blvd., Campus Box 8225, Saint Louis, MO 63110 USA; Division of Biology and Biomedical Sciences, Washington University in St. Louis, 510 South Kingshighway Blvd., Campus Box 8225, Saint Louis, MO 63110 USA

**Keywords:** Sex differences, Lipid metabolism, Acylcarnitines, Triglycerides, NEFA, Type 2 diabetes, Non-obese, Lipidomics, Genomics, Cardiac metabolism, Biomarker, ROS

## Abstract

**Background:**

Cardiovascular disease is the leading cause of death among diabetic patients. Importantly, recent data highlight the apparent sexual dimorphism in the pathogenesis of cardiovascular disease in diabetics with respect to both frequency- and age-related risk factors. The disposition to cardiovascular disease among diabetic patients has been attributed, at least in part, to excess lipid supply to the heart culminating in lipotoxicity of the heart and downstream derangements. A confounding factor in obese animal models of diabetes is that increased peripheral lipid availability to the heart can induce cardio-metabolic remodeling independent of the underlying pathophysiology of diabetes, thus masking the diabetic phenotype. To that end, we hypothesized that the use of non-obese diabetic (NOD) animal models will reveal metabolic signatures of diabetes in a sex-specific manner.

**Methods:**

To test this hypothesis, male and female NOD Goto-Kakizaki (GK) rats were used to assess the expression profile of 84 genes involved in lipid metabolism. In parallel, targeted lipidomics analysis was performed to characterize sex differences in homeostasis of non-esterified fatty acids (NEFA), acylcarnitines (AC), and triglycerides (TG).

**Results:**

Our analysis revealed significant sex differences in the expression of a broad range of genes involved in transport, activation, and utilization of lipids. Furthermore, NOD male rats exhibited enhanced oxidative metabolism and accumulation of TG, whereas female NOD rats exhibited reduced TG content coupled with accumulation of AC species. Multi-dimensional statistical analysis identified saturated AC16:0, AC18:0, and AC20:0 as dominant metabolites in mediating sex differences in AC metabolism. Confocal microscopy of rat cardiomyocytes exposed to AC14:0, AC16:0, and AC18:0 confirmed induction of ROS with AC18:0 being more potent followed by AC14:0.

**Conclusion:**

Overall, we demonstrate sex differences in myocardial AC and TG metabolism with implications for therapy and diagnosis of diabetic cardiovascular disease.

**Electronic supplementary material:**

The online version of this article (doi:10.1186/s13293-016-0077-7) contains supplementary material, which is available to authorized users.

## Background

Heart disease is the leading cause of death among diabetic patients, independent of macro- and micro- vascular diseases [1–3]. While diabetes increases the risk of cardiovascular disease in both males and females, recent data suggests that heart disease is fivefold more common in diabetic women compared with twofold in men [4]. For example, in the setting of myocardial infarction, diabetic women have a greater progression to heart failure and poorer outcome compared with diabetic males [5]. This “sexual dimorphism” is in contrast to the established notion of a “female advantage” in that premenopausal women in general are better protected from most common forms of cardiovascular disease compared to men [4, 6–9]. Thus, T2DM nullifies the female advantage in the prevalence of cardiovascular disease [4, 10].

Multiple lines of evidence suggest that the predisposition to heart failure among diabetic patients is a consequence of severe alterations in myocardial substrate metabolism [11, 12]. In particular, insulin resistance shifts the balance of substrate utilization such that the diabetic heart relies almost exclusively on fatty acids (FAs) for its energy needs [13]. The increased reliance on FA oxidation competes with glucose oxidation further exacerbating deficiencies in insulin signaling [14]. Excess lipid supply beyond the oxidative capacity of the heart results in accumulation of triglycerides (TG) and lipid intermediates, leading to lipotoxicity of the heart and downstream derangements, including generation of reactive oxygen species (ROS), inflammation, and cell death [15–20].

On the one hand, the observed sexual dimorphism may be explained by the higher systemic availability of lipids [21, 22] resulting in increased myocardial FA metabolism. Indeed, it is well established that females have higher distribution of visceral fat with higher levels of circulating FA and TGs [22]. As an omnivore, the heart will necessarily utilize circulating substrates in proportion to their availability in blood. To that end, in vivo metabolic imaging studies in diabetic humans have demonstrated that, in fact, female hearts exhibit higher flux of FAs compared to age-matched males [23, 24], attributed to higher systemic availability of FA. On the other hand, however, there may be intrinsic sex differences in cardiac lipid metabolism beyond increased peripheral supply of lipids to the heart that may account for the sexual dimorphism. One confounding factor in obese and dietary animal models of diabetes is that increased peripheral lipid availability to the heart can induce cardio-metabolic remodeling independent of the underlying pathophysiology of diabetes, thus masking the diabetic phenotype. In this regard, it is worth noting that, in fact, 20 % of the population is non-obese diabetic (NOD) [25, 26]. This suggests that studies utilizing NOD humans and/or non-obese animal models of diabetes may unmask mechanisms relevant to pathophysiology of diabetes.

In this work, we sought to determine whether there are intrinsic sex differences in myocardial FA metabolism in the setting of diabetes, independent of systemic effects seen in obesity or dietary animal models of diabetes. We previously demonstrated that NOD Goto-Kakizaki (GK) rats exhibit a genomic disposition to increased FA metabolism [27]. We hypothesized that utilizing both sexes of this model will reveal metabolic signatures of diabetes independent of obesity, in a sex-specific manner. Quantitative expression profiling of genes involved in FA, acylcarnitines (AC), and TG metabolism as well as targeted lipidomic profiling on cardiac tissue extracts was performed to characterize genomic-metabolic variations between the groups. Finally, multi-dimensional statistical analysis was performed to identify and confirm signatures of sex differences in metabolism that can induce ROS.

## Methods

### Animal model and study design

All experiments were conducted according to the protocol approved by the animal experiment committee at Washington University School of Medicine at Saint Louis (IACUC Animal Welfare Assurance # A-3381-01). NOD and Wistar rats (Charles River, USA) were maintained under standard housing conditions and fed with commercial pelleted chow (Harlan, USA) until ready for the study at age of 24 weeks. A balanced factorial design at two levels was used to investigate sex and phenotype differences and possible interactions between these two factors. The following four animal groups were tested: (1) Wistar male, (2) Wistar female, (3) GK male, and (4) GK female with *N* = 4–6 per group. Blood samples were taken via the tail vein from anesthetized animals (overnight fast, typically 10 h) in each group and immediately centrifuged. The serum fractions were assayed for TG, non-esterified fatty acids (NEFA), glucose, and insulin levels. All animals were sacrificed at the end of the study, and their hearts were snap-frozen for subsequent analyses. Lipidomic analysis was performed on individual tissue extracts (*N* = 6 per group), while gene expression studies was performed on representative samples (*N* = 4 per group), randomly chosen from each group. FA and TG were extracted from 100 mg of pulverized cardiac tissue samples using a chloroform/methanol mixture. Commercial kits for FA (Wako Diagnostics, VA) and TG analyses (Infinity Triglycerides Reagent, Thermo Scientific, USA) were used for both plasma and tissue samples.

### RNA isolation and real-time RT-PCR

Quantitative real-time RT-PCR (qPCR) was performed as described previously [27]. The RNeasy lipid tissue extraction kit (Qiagen, MD) was used to extract total RNA from pulverized heart tissue. RNA concentration, purity, and integrity were determined by spectrophotometric analysis and Agilent Bio-analyzer. qPCR was conducted in a 384-well plate array format for 84 genes involved in FA metabolism (Cat No: PARN- 0007E, SA Biosciences, MD). From the panel of housekeeping genes, lactate dehydrogenase A (*ldhA*) was chosen for normalizing the data as it displayed no variation among test groups. For assessing ROS response in AC-treated cardiomyocytes, PrimeTime® primers for *Sod1*, *Sod2*, and *Cat* were sourced from IDT DNA technologies (Coralville, IA).

### Mass spectrometric detection of NEFAs, ACs, and TGs

Mass spectrometric analysis was performed at the Diabetic Cardiovascular Disease Center (DCDC) Lipidomics Core Facility using established methodology. Briefly, a modified Bligh-Dyer extraction [28] was used to extract NEFAs and TGs, and a protein precipitation method was used to extract ACs from the heart homogenates in the presence of internal standards (d4-NEFA16:0, TG17:1-17:1-17:1, and d3-AC16:0). To improve the sensitivity of MS, NEFAs were derivatized with dimethylaminopropylamine, dimethylaminopyridine, and 1-ethyl-3-[3-dimethylaminopropyl] carbodiimide hydrochloride into amides, and ACs were derivatized with methanol and acetyl chloride into methyl esters. Sample analysis was performed with a Shimadzu 10A HPLC system coupled to a TSQ Quantum Ultra triple quadrupole mass spectrometer operated in SRM mode under ESI(+).

### Echocardiography

Echocardiography was performed using non-invasive ultrasound imaging with the Vevo2100 Ultrasound System (Visual Sonics Inc., Toronto, ON, Canada) at ages 23–24 weeks as described previously [29]. Briefly, rats were anesthetized with 1 % Isoflurane and secured to the imaging platform. Complete two-dimensional, M-mode, and Doppler examinations using a 21-MHz transducer were performed to quantify left ventricular structure as well as diastolic and systolic function. To account for differences in body weight, all of the structural dimensions were indexed to body weight.

### Confocal microscopy to assess induction of ROS by ACs

Immortalized neonatal rat cardiomyocytes were obtained from Applied Biological Materials, Inc. Canada (Catalog No. T0015) and maintained in PriGrow III media (ABM Good, Canada). The method for microscopic determination of ROS has been described previously [30, 31] and was used here with minor modifications appropriate for this study. Briefly, cells were seeded at 1 × 10^5^ per 35-mm glass-bottomed μdish (Catalog no. 81158 iBidi, Germany) and incubated overnight. The cells were serum starved for 8 h and then treated with 10 μM CM-H_2_DCFDA (Molecular Probes, C6827), a chloromethyl (CM) derivative of H_2_DCFDA, in PBS at 37 °C for 30 min. The treatment was followed by two quick washes using ice cold PBS. Based on previous work [30], cells were incubated with 25 μM of each of the following ACs for 3 h at 37 °C: palmitoyl-l-carnitine (AC16:0) (P1645, Sigma-Aldrich), stearoyl-l-carnitine (AC18:0) (50494231, Fisher Scientific), and myristoyl-l-carnitine (AC14:0) (41570, Crystal Chem, IL). The 3-h incubation period was selected to remain within the stability range of the fluorophore per manufacturer’s recommendation. Cells were then examined using a Nikon A1Rsi + laser scanning confocal microscope at ×40 magnification using laser excitation at 488 and emission 543 nm at the Bakewell Neuroimaging Laboratory at Washington University. ROIs were defined using NIS-Elements (Nikon, USA) on 30–35 images by filtering background and saturated intensities as well as filtering for objects/debris that were less than 5 μm in size as rat cardiomyocytes are at least 10 μm in size [32]. The sum intensity of ROIs for each image from the test groups were compared with the control by *t* test.

### Data and statistical analysis

All data sets from the two level factorial designs were first examined to verify equal variance among test groups using residual analysis, and if necessary, the data were log_2_ transformed. The data were analyzed using a two-way ANOVA with interaction model (sex, phenotype, sex*phenotype). ANOVA was followed with group contrast comparisons using Fisher’s least squares difference to determine significance. Student’s *t* test was used to determine significant statistical differences in fluorescence intensities obtained from our microscopic analysis of genes *Sod1*, *Sod2*, and *Cat* between control and AC-treated groups.

#### Sex differences in the distribution of lipid species

We also sought to assess whether the fraction (as percent) of species enriched/depleted in each lipid category (i.e., FA, TG, and AC) was significantly different within and between sexes. This analysis ignored the statistical findings of the ANOVA for individual species; rather, the number of species enriched or depleted in the respective category was counted. The significance of the overall fold change in the number of species enriched or depleted (GK to Wistar) profile was determined by comparing to a binomial distribution with the assumption that if the variable had no effect, then the number of species enriched or depleted would be random with a binomial distribution with mean = 0.5.

#### Sex differences in metabolic pathways

Genes were categorized into the metabolic functional groups. Because the interpretation of the ANOVA interaction term for the genes was more nuanced than for the metabolites (i.e., many interactions were due to differences in magnitude rather than just sign), a rank-sum test, which considers both sign and magnitude, was used to determine significant differences in metabolic pathways between the sexes. The GK/Wistar fold change sign and magnitude was used as the test parameter and sex was the categorical variable. The data were analyzed for low-expressing genes using a criterion that the Ct count must not be greater than 2 standard deviations from the average of all genes. Therefore, any gene with a Ct count >28 was considered non-expressing.

#### Principal component analysis

Principal component analysis (PCA) was performed on the gene and metabolite data separately using the correlation matrix of the standardized data. Only those genes or metabolites that had at least one significant contrast comparison in the ANOVA analysis were included in the PCA. As in above, low-expressing genes were omitted from the analysis. Radar plots were used to present the PCA results. Partek Genomics Suite (St. Louis, MO) was used for ANOVA, STATA (College Station, TX) for the binomial distribution tests, and JMP Genomics for the PCA. The data are presented as mean ± standard error of the mean (SEM) and a *P* value ≤0.05 was considered significant. While the statistics may have been performed on transformed data, the results are graphed in their original metric as a convenience to the reader.

## Results

### Baseline characteristics

We found no significant differences in serum FA level among the four groups. Serum TG levels were significantly elevated in NOD rats with males and females exhibiting 1.38- and 2.2-fold increase over corresponding Wistar rats, respectively (Table 1). Overall, these baseline characteristics are in agreement with previous reports on GK rats [33, 34].

**Table 1:** Baseline measurements

	Male		Female		ANOVA		
	Wistar	GK	Wistar	GK	Phenotype	Sex	Phen × sex
	(*N* = 4)	(*N* = 4)	(*N* = 4)	(*N* = 4)			
Weight (g)	491.67 ± 5.34	334.17 ± 10.26*	256.97 ± 5.35	230 ± 2.80^‡^	*<0.001*	*<0.001*	*<0.001*
Insulin (μU/mL)	14.5 ± 5.76	12.33 ± 2.00	11.86 ± 3.56	7.46 1.37	0.199	0.348	0.362
Glucose (mmol/L)	7.80 ± 0.43	9.85 ± 0.54^†^	7.68 ± 0.57	9.76 ± 1.25^†^	*0.020*	.0936	0.745
Serum NEFA (μmol/L)	1084.50 ± 155.94	1434.25 ± 124.64	1421.75 ± 202.11	1406.25 ± 130.01	0.305	0.341	0.265
Serum TG (μmol/L)	877.01 ± 155.69	1219.97 ± 119.37^‡^	525.78 ± 88.95	1157.80 ± 244.44^‡^	*0.011*	0.228	0.392
Tissue NEFA (μg/mg)	0.77 ± 0.03	1.34 ± 0.18^†^	0.74 ± 0.04	0.98 ± 0.06	*0.0005*	0.071	1

Values are mean ± SEM. Italicized values indicate significance at *P* < 0.05, ^†^ *P* < 0.05, ^‡^ *P* < 0.01, *P < 0.001 GK significantly different than Wistars

*GK* Goto-Kakizaki rats

### Echocardiograph: anatomical and functional parameters

Weight-indexed measures of LVPW, LVID, and LVM were significantly higher in NOD rats compared to corresponding Wistar control rats (Table 2). Functionally, NOD rats exhibited a significant reduction in parameters of early component mitral annular diastolic motion (E′) and LV filling velocity in the atrial component (A) compared to Wistars, as well as both phenotypic and sex differences in early left ventricular filling velocity (E) (Table 2).

**Table 2:** Echocardiograph measurements

	Male		Female		ANOVA		
Parameter	Wistar	GK	Wistar	GK	Phenotype	Sex	Phenotype × sex
HR (bpm)	396.22 ± 16.90	345.56 ± 27.34*	388.13 ± 16.36	324.67 ± 25.41‡	*<0.001*	0.343	0.67
LVPWId (μm/g)	0.0037 ± 0.0003	0.0049 ± 0.0005	0.0046 ± 0.0003	0.0063 ± 0.0002	*<0.0005*	*<0.0005*	0.31
LVPWIs (μm/g)	0.0060 ± 0.0003	0.0073 ± 0.0006†	0.0083 ± 0.0005	0.0102 ± 0.0008	*<0.0005*	*<0.0005*	0.49
LVIDId (μm/g)	0.0150 ± 0.001	0.0219 ± 0.001	0.0240 ± 0.001	0.0291 ± 0.001‡	*<0.0005*	*<0.0005*	0.35
LVIDIs (μm/g)	0.0087 ± 0.0007	0.0136 ± 0.0013	0.0120 ± 0.0013	0.0170 ± 0.0009	*<0.0005*	*<0.0005*	0.82
LVMI (mg/g)	2.45 ± 0.15	2.84 ± 0.18*	2.52 ± 0.10	3.04 ± 0.19*	*<0.0005*	0.23	0.54
FS (%)	43.45 ± 1.78	38.20 ± 4.44	48.52 ± 3.91	41.57 ± 3.45	*<0.05*	0.092	0.728
E (mm/s)	1041.33 ± 82.04	818.50 ± 39.05*	838.67 ± 49.27	750.57 ± 48.21*	*<0.01*	*<0.01*	0.161
E′ (mm/s)	54.43 ± 8.10	36.26 ± 6.77*	44.65 ± 3.23	30.04 ± 5.51‡	*<0.01*	*0.124*	0.725
A (mm/s)	813.67 ± 76.65	560.50 ± 38.94*	727.50 ± 57.27	535.43 ± 48.99‡	*<0.0005*	*0.257*	0.528
A′ (mm/s)	51.35 ± 6.4	52.27 ± 5.97	62.67 ± 5.71	55.24 ± 6.07	*0.527*	*0.173*	0.419

Values are mean ± SEM. Italicized values indicate significance at *P* < 0.05. *P < 0.05, ‡*P* < 0.01, †*P* < 0.001 GK significantly different than Wistars

*GK* Goto-Kakizaki rats, *HR* heart rate, *LVPWId and LVPWIs* left ventricle posterior wall index during diastole and systole, respectively, *LVIDId and LVIDIs* left ventricle internal dimension index during diastole and systole, respectively, *LVMI* Left ventricle mass index as measure of cardiac structural parameters, *FS* fractional shortening, *E, E′, A, A′* measures of transmitral velocity

### Sex differences in expression profile of genes involved in lipid metabolism

**Fig. 1:**
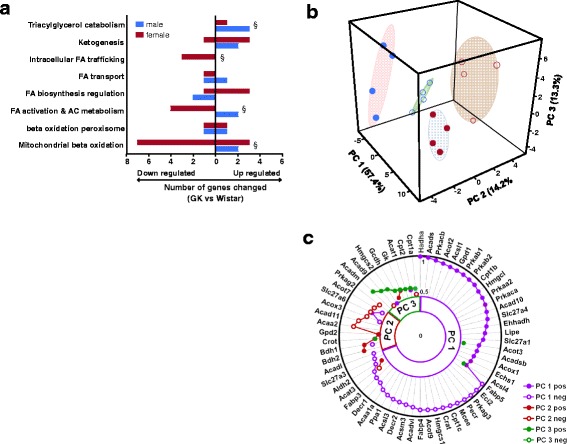
Sex differences in expression profile of genes involved in lipid metabolism. **a** Number of genes modulated between NOD and Wistar rats by sex from ANOVA model. A section sign (§) indicates that pathways differ between sexes by the rank-sum test (*P* ≤ 0.05). **b** 3D score plot of first three principal components (PCs) for genes from principal component analysis. **c** Shows the radar plot of loading factors of PCs for gene analysis. The *purple line* and *circles* represent PC1, the *brown line* and *circles* represent PC2, and *green line* and *circles* represent PC3. While absolute loading values are used to plot, the *closed* and *open circles* represent (+) and (–) loading values, respectively. *P* < 0.05 is the significance level for all tests

Figure 1a summarizes the ANOVA results for the differential expression of genes categorized by their metabolic functionality. The number of genes modulated between NOD and Wistar by sex is shown for each functional group. Overall, the pathway analysis indicates a significant increase in the expression of genes involved in beta-oxidation in NOD male rats relative to female rats, concomitant with reduced expression of genes involved FA activation and AC metabolism in NOD female rats. Other significant sex differences in pathway expression include intracellular FA trafficking and triacylglycerol metabolism. To characterize which genes dominate the variance among the groups, we performed PCA to reduce the dimensionality of the data.

Figure 1b depicts the segregation of the four test groups by PCA. The first three principal components (PCs) accounted for 85 % of the variability. Figure 1c is a radar plot of absolute gene loading values greater than 0.5 for each PCs. The genes were sorted by decreasing order of the absolute magnitude of their primary component. The PCs are associated with general fold change patterns that can be discerned from examination of Additional file 1: Table S1. PC1 accounts for 57.4 % of the variation in the data and is associated with genes that exhibit sex differences and a significant interaction term in the ANOVA model. Those genes with a positive PC1 value (PC1pos) are downregulated in females compared to males, while those with a negative value (PC1neg) are upregulated in females. Further, the interaction is far more prevalent in genes associated with PC1neg in that the sex difference fold changes are higher in Wistar than in the NOD phenotype. Generally, genes associated with PC2(+) are elevated in NOD rats, while those with PC2(−) are lower in NOD rats. PC3 has too few genes to ascribe a pattern. Overall, the loading values can be used to correlate the contribution of a gene/pathway to the overall phenotype of sex differences in metabolism.

### Sex differences in NEFA homeostasis

**Fig. 2:**
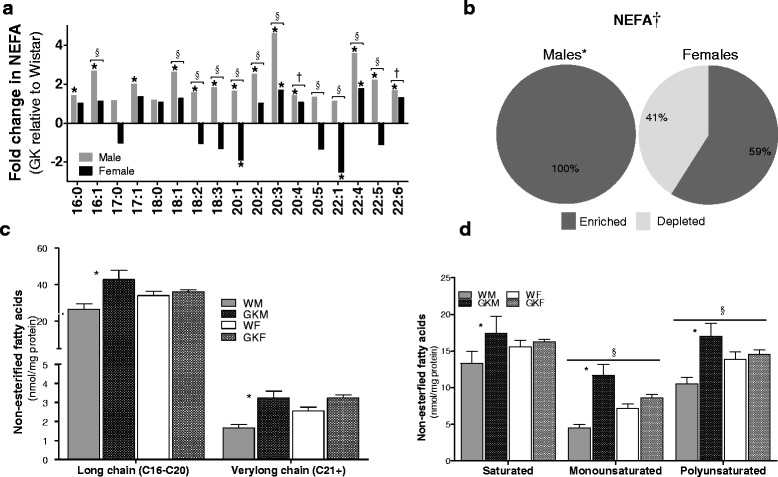
Sex differences in NEFA homeostasis. **a** Profile of NEFA in cardiac tissue, as measured by LC-MS/MS, presented as fold change between NOD and Wistar rats by sex. An asterisk (*) indicates that the fold change is statistically significant. A section sign (§) indicates that the ANOVA interaction term is significant, and therefore, the fold changes in males and females also are. A dagger (†) indicates that in the ANOVA model, the overall sex difference is statistically significant. **b** Distribution of enriched and depleted of NEFA moities. The asterisk (*) indicates that the distribution is significantly different than a binomial distribution with mean = 0.5. The dagger (†) indicates that the distribution in males and females differ significantly. **c**, **d** The distribution of FAs based on their chain-length and degree of saturation, respectively. Data is presented as mean ± SEM (*N* = 6). An asterisk (*) indicates that the difference in adjacent bars is statistically significant. A section sign (§) indicates that the ANOVA interaction term is significant. *P* < 0.05 is the significance level for all tests

The enrichment profile of NEFAs obtained by LC-MS/MS is summarized in Fig. 2a (Additional file 2: Figure S1 depicts male and female data separately). Our results reveal that of the NEFAs profiled, every measured NEFA species is significantly enriched in male NOD rats compared to male Wistar rats in agreement with overall tissue NEFA concentration summarized in Table 1. In females, however, only NEFAs 20:1 and 22:1 are significantly depleted in NOD, while NEFAs 20:3 and 22:4 are significantly enriched in NODs. Based on the binomial distribution analysis, there is a significant sex difference in the distribution of NEFAs with male NOD exhibiting significant enrichment of lipid species (Fig. 2b). The data in Fig. 2c suggests that the concentration of both long and very long NEFAs is higher in NOD compared to Wistar rats, but the differences only reach statistical significance in males. Analysis of NEFA profile based on degree of saturation reveals that saturated, mono-, and poly-unsaturated fatty acids accumulate to a higher extent in NOD compared with Wistar rats, but the differences are only significant in males (Fig. 2d). Furthermore, NOD rats overall exhibit significant accumulation of mono- and poly-unsaturated fatty acids compared to Wistar rats.

### Sex differences in TG homeostasis

**Fig. 3:**
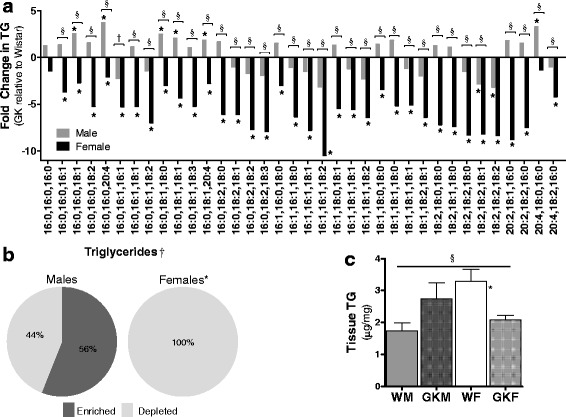
Sex differences in TG homeostasis. **a** Profile of TG species in cardiac tissue, as measured by LC-MS/MS, presented as fold change between NOD and Wistar rats by sex. An asterisk (*) indicates that the fold change is statistically significant. A section sign (§) indicates that the ANOVA interaction term is significant, and therefore, the fold changes in males and females are also significant. A dagger (†) indicates that in the ANOVA model, the overall sex difference is statistically significant. **b** Distribution of enriched and depleted of TG moities. The asterisk (*) indicates that the distribution is significantly different than a binomial distribution with mean = 0.5. The dagger (†) indicates that the distribution in males and females differ significantly. **c** The total amount of TG present in the cardiac tissue as measured by spectrophotometric analysis, mean ± SEM (*N* = 6). *P* < 0.05 is the significance level for all tests

Analysis of the individual TG species levels by LC-MS/MS indicates that all measured TG species were depleted in NOD females compared to Wistar females (Fig. 3a) (Additional file 3: Figure S2 depicts male and female data separately). Moreover, the binomial distribution analysis suggests that the number of species depleted in females was statistically significant, compared to males (see “Methods” section) (Fig. 3b). Consistent with these data, the spectrophotometric analysis of total TG in cardiac tissue suggests that female NOD rats exhibited significantly reduced TG concentrations than the Wistar female, while male NOD rats exhibited a higher average TG content. Although the difference in males is not significant, the significant interaction suggests that the differences in total TG accumulation between NOD and Wistar rats differs significantly in males and females (Fig. 3c).

### Sex differences in AC homeostasis

**Fig. 4:**
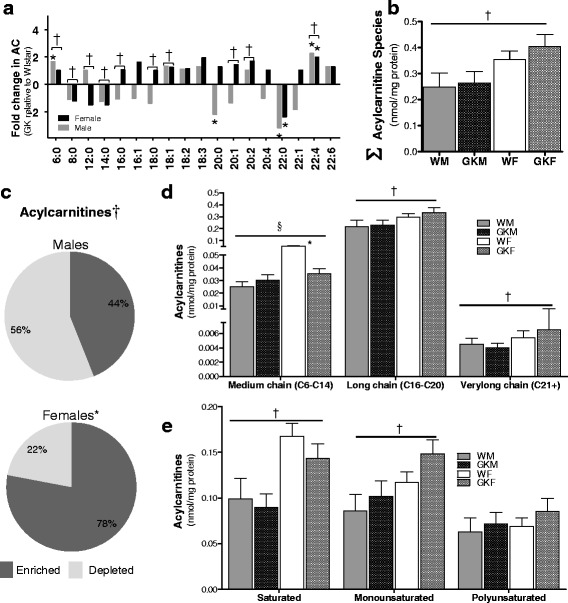
Sex differences in AC homeostasis. **a** Profile of AC in cardiac tissue, as measured by LC-MS/MS, presented as fold change between NOD and Wistar rats by sex. An asterisk (*) indicates that the fold change is statistically significant. A dagger (†) indicates that in the ANOVA model, the overall sex difference is statistically significant. **b** Net ACs level in the tissue, identified by summing up individual AC species. The dagger (†) indicates that in the ANOVA model the overall sex difference is statistically significant. **c** Distribution of enriched and depleted of AC moities. The asterisk (*) indicates that the distribution is significantly different than a binomial distribution with mean = 0.5. The dagger (†) indicates that the distribution in males and females differ significantly. **d** Pooled distribution of ACs based on their chain length. **e** Pooled distribution of ACs based degree of saturation. Data in **d** and **e** are presented as mean ± SEM (*N* = 6). The asterisk (*) indicates that comparison between adjacent bars is significant. The section sign (§) indicates that the ANOVA interaction term is significant. A dagger (†) indicates that in the ANOVA model, the overall sex difference is statistically significant

The LC-MS/MS metabolic profile of ACs revealed significant sex differences in the ACs profiled (Fig. 4a) (Additional file 4: Figure S3 depicts male and female data separately). Moreover, our data suggests sex differences in abundance of ACs with female rats exhibiting significantly higher abundance of AC (Fig. 4b) and species (Fig. 4c) compared to male rats. Since tissue ACs are elevated in female rats, we sought to determine whether differences in serum TG or NEFA levels could account for the higher AC levels in tissue. To test this possibility, we normalized the AC concentration to the sum of the serum TG and NEFA concentrations for each rat and ran the ANOVA model on this modified data. Our data (not shown) indicate sex differences in AC levels independent of serum TG and NEFA. Thus, the elevated serum levels cannot sufficiently account for the increased AC levels in female.

The AC concentration data was binned by medium, long, and very long chains, and the results plotted in Fig. 4d. The results indicate that AC concentrations are lower in males for all chain lengths and that for the medium chain lengths, the concentrations are statistically lower in NOD females than in Wistar females. These observations are consistent with the information in Fig. 4a, b. It is also observed from Fig. 4a that, for the short chain AC, 4/4 are elevated in females in Wistar rats, while only the longest of the short chain AC is statistically elevated in NOD females compared to males. In the long chain AC, however, 5/10 are statistically higher in female compared to male NOD rats, while only 1/10 is statistically higher in Wistar rats. Finally, partitioning of AC by degree of saturation suggests that female rats exhibit higher levels of saturated and mono-unsaturated AC (Fig. 4e).

### Integrated lipidomics analysis of NEFA, AC, and TG

**Fig. 5:**
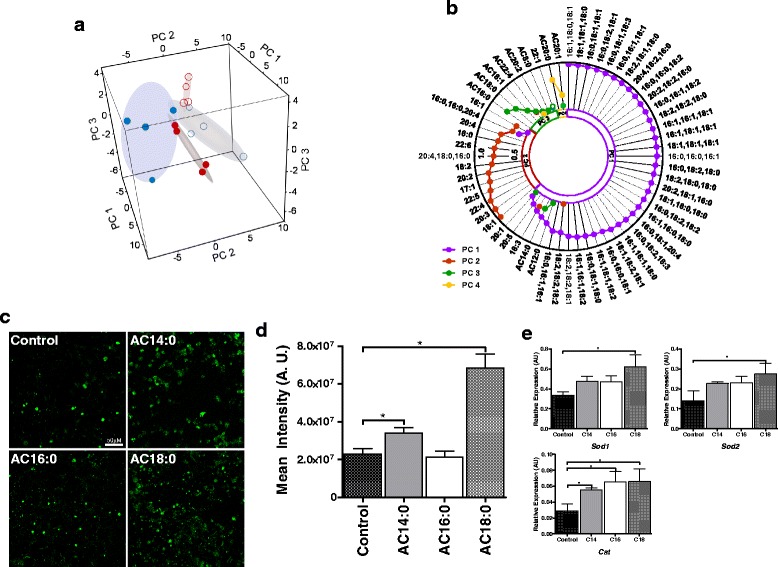
Integrated lipidomics analysis of NEFA, AC, and TG and induction of ROS by ACs. **a** 3D score plot of first three principal components (PCs) for metabolites obtained from PCA. **b** Radar plot of loading factors of principal components for lipidomic analysis. The *purple line* and *circles* represent PC1, the *brown line* and *circles* represent PC2, the *green line* and *circles* represent PC3, and the *yellow line* and *circle* represents PC4. All loadings were positive and hence are depicted using *closed circles*. **c** Depicted are representative confocal microscopy images of cardiomyocytes incubated with ethanol (control) and 25 μM of AC14:0, AC16:0, and AC18:0 (*bar* = 50 μM). **d** The sum intensities of each image from the test groups were compared with untreated cells. Averages of the image intensities, mean + SEM are shown. **e** Relative expression levels of genes *Sod1*, *Sod2*, and *Cat* in cardiomyocytes compared to the untreated control cells. Average C_T_ of *b-actin* and *LdhA* was used to normalize the data. An asterisk (*) indicates that the comparison identified by the *bar* is significant at *P* < 0.05

Figure 5a reveals the segregation of the lipidomics data among the four test groups based on PCA analysis. Figure 5b summarizes the results of the PCA and is a radar plot of absolute gene loading values greater than 0.5 for each PC. The general fold change pattern associated with each PC can be discerned from examination of Additional file 5: Table S2. PC1 accounts for 52.4 % of the variation in the data and is associated almost exclusively with TG that are elevated in female Wistar over male Wistars. There are two AC associated with PC1, and both are elevated in females irrespective of phenotype. There are also three NEFAs associated with PC1 that exhibit interactions both with respect to female vs male by phenotype and GK vs Wistar by sex.

PC2 accounts for 20.7 % of the variation and is associated primarily with NEFA. PC2 species also have significant interaction terms in their ANOVA models. NEFA are elevated in females among Wistar rats but are depleted in females among NOD rats. Among males, NEFA are elevated in NOD rats, while generally the same among females. PC3 is composed primarily of ACs, which are elevated in females irrespective of phenotype. One AC, 22:4, is also elevated in NOD over Wistar overall. PC4 is also exclusively AC of 20 carbon length but differs from PC3 in that the interaction term is significant in the ANOVA model. AC 20:0 is elevated in females among NOD rats but is unchanged with sex in Wistar, and it is depleted in NOD among male rats.

Of the FA examined, PCA identified FA 18:1, 20:3, and 22:4 as having the most influence in dictating sex differences in FA metabolism, whereas AC16:0 and AC18:0 in PC3 as well as AC20:0 in PC4 were identified as contributing to sex differences in AC metabolism (Fig. 5b). To test the feasibility of the former set of ACs to induce ROS, cardiomyocytes were exposed to 25 μM of each AC14:0, AC16:0, and AC18:0 ACs. Quantitative analysis of confocal microscopy images (Fig. 5c) suggests that the relative efficiency of ROS induction was AC18:0 > AC14:0 > AC16:0 (Fig. 5d). Accordingly, the expression levels of *Sod1*, *Sod2*, and *Cat* genes encoding for key enzymes involved in ROS scavenging were significantly higher in AC-treated cardiomyocytes (Fig. 5e). In agreement with quantitative confocal microscopy images, the magnitude of differential expression was highest in AC18:0-treated cardiomyocytes compared to untreated controls (Fig. 5e).

## Discussion

The overarching objective of this work was to identify sex differences in lipid metabolism in a NOD animal model of T2DM. This effort was partly motivated by the lack of adequate animal models to study sex differences in T2DM [35]. Since gene expression alone may be insufficient to address the question of sex differences in metabolism, we pursed a genomic-metabolomic approach to characterize sex differences in metabolism where changes in metabolite species are the ultimate end-product of gene and protein expression differences. Of note, it is well established that women generally have a higher percentage of body fat than men [36], resulting in increased levels of circulating FA and TGs. Since increased peripheral lipid availability to the heart, as is the case in obesity, can induce cardio-metabolic remodeling independent of the underlying pathophysiology of diabetes, we hypothesized that the use of NOD animal models of T2DM will reveal metabolic signatures of diabetes, in a sex-specific manner.

Unlike obese animal models of T2DM, NOD rats did not display significant differences in circulating NEFA levels although they displayed slightly elevated TG, consistent with previous work on NOD rats [37–40]. In cardiac tissue, NOD rats exhibited elevated steady state levels of NEFAs, which was particularly higher in male NOD rats. Expression analysis of gene arrays involved in FA metabolism revealed marked differences in mitochondrial beta-oxidation, most notably preference towards FA oxidation in male NOD rats in contrast to down-regulation of gene involved in FA oxidation in female rats (Fig. 1). In parallel, we observed significant sex differences in the expression of genes involved in FA activation and AC metabolism. Moreover, multi-dimensional PCA analysis identified significant differences in the expression levels of a broad set of genes regulating multiple pathways including energy sensing, FA catabolism, acylcarnitine transport and activation, and that of TG biosynthesis and degradation. To assess downstream implications of the differences in gene expression, we performed lipidomic profiling of NEFA, TG, and AC in cardiac tissues.

Our lipidomics data suggests sex differences in TG homeostasis. NOD female rats exhibited significantly lower total TG concentrations than female Wistar rats, and virtually all the TG species assayed were lower in NOD females compared with age-matched Wistar rats. While TGs have long been proposed to adversely affect cardiac function and physiology [16, 18], recent data suggests that TG may also play a cardio-protective role by absorbing excess lipid supply to the heart [41, 42]. The observation that female NOD hearts display significantly lower levels of TG may suggest that FAs were channeled away from TG synthesis or that TGs were converted into other lipid products such as DAG and ceramides.

In addition to sex differences in TG metabolism, our lipidomic analysis also revealed sex differences in AC metabolism with female rats exhibiting higher concentration of ACs than male rats. Long-chain ACs have long ago been documented as arrhythmogenic affecting myocyte electrophysiology [43–45] and influencing recovery following ischemic injury [46, 47]. However, recent mechanistic [48, 49] and metabolomic [50–53] studies using diabetic animal models have identified intra-mitochondrial disturbances where lipid overload resulted in the accumulation of AC and incomplete FA oxidation [54]. The role of AC in T2DM has been highlighted by recent reports suggesting that serum levels of ACs are altered in pre-diabetic conditions [54, 55] and by reports identifying serum AC species indicative of enhanced risk to T2DM and insulin resistance [31, 56–59]. Importantly, accumulation of ACs has been shown to interfere with insulin signaling [48, 60–63], activate pro-inflammatory signaling pathways [30], induce cellular uncoupling [64], and generate cytoplasmic ROS [30] in murine monocyte/macrophages.

In the current work, PC3 in Fig. 5c identified AC16:0 and AC18:0 as having the most variability in dictating sex differences in AC metabolism. To test the feasibility of AC to induce ROS in cardiomyocytes, rat cardiomyocytes were exposed to 25 μM of AC16:0, AC18:0, and AC14:0 (the latter based on literature precedent [30]). Our results suggest that AC18 induced over threefold higher production of ROS than untreated cardiomyocytes, followed by AC14:0. In parallel to increased ROS as a response to AC stimuli, expression analysis of genes with established roles in ROS scavenging confirmed activation of ROS mitigating pathways. These observations demonstrate, for the first time, that ACs can induce production of ROS in cardiomyocytes. Further studies are required to fully characterize the specificity and selectivity of ACs in inducing cardiac ROS, insulin resistance, and downstream derangements in cardiomyocyte health and function in a sex-specific manner.

## Conclusions

In summary, these observations represent the first such report of sex differences in AC and TG metabolism as a potential metabolic signature of diabetes, which may contribute to downstream derangements in cardiac function in a sex-specific manner. Our analysis revealed sex differences in the expression of a broad range of genes involved in the metabolism of FAs, ACs, and TGs. Furthermore, integrated lipidomics analysis indicated that whereas NOD male rats exhibit enhanced oxidative metabolism activity and accumulation of TG species, female NOD rats tend to exhibit enhanced accumulation of AC species and reduced TG compared to male rats. Finally, we have demonstrated that ACs can induce production of ROS in cardiomyocytes, providing a new dimension for the observed sexual dimorphism in diabetic cardiovascular disease. Some of the potential implications include the following: from a therapeutic point, therapies that minimize accumulation of ACs and potentially other by-products of beta-oxidation may be of interest in treating females. From an imaging standpoint, our data suggests that currently available metabolic tracers, in particular for positron emission tomography (PET), may be unable to discern intrinsic (as opposed to systemic) sex differences in lipid metabolism, suggesting that new panel of metabolic PET tracers may be needed to fully interrogate sex differences in cardiac metabolism and downstream effects. Thus, overall, our data highlight broad implications for therapy and diagnosis of T2DM.

## Additional files

Additional file 1: Table S1. Summary statistics, ANOVA and principal component analysis results for genes. (XLSX 31 kb) Additional file 2: Figure S1. Concentrations of non-esterified fatty acid moieties in NOD and Wistar rats by sex. (PPTX 163 kb) Additional file 3: Figure S2. Concentrations of triglyceride moieties in NOD and Wistar rats by sex. (PPTX 226 kb) Additional file 4: Figure S3. Concentrations of acylcarnitine moieties in NOD and Wistar rats by sex. (PPTX 142 kb) Additional file 5: Table S2. Summary statistics and ANOVA and principal component analysis results for metabolites. (XLSX 43 kb)
