# Supplementary material for: Sexual dimorphism in myocardial acylcarnitine and triglyceride metabolism
Source: Biol Sex Differ. 2016 May 13;7:25. doi: 10.1186/s13293-016-0077-7 (PMC4866274; doi:10.1186/s13293-016-0077-7)
Supplement: Additional file 3: — Figure S2. Concentrations of triglyceride moieties in NOD and Wistar rats by sex. (PPTX 226 kb) [file 13293_2016_77_MOESM3_ESM.pptx]

## Slide 1
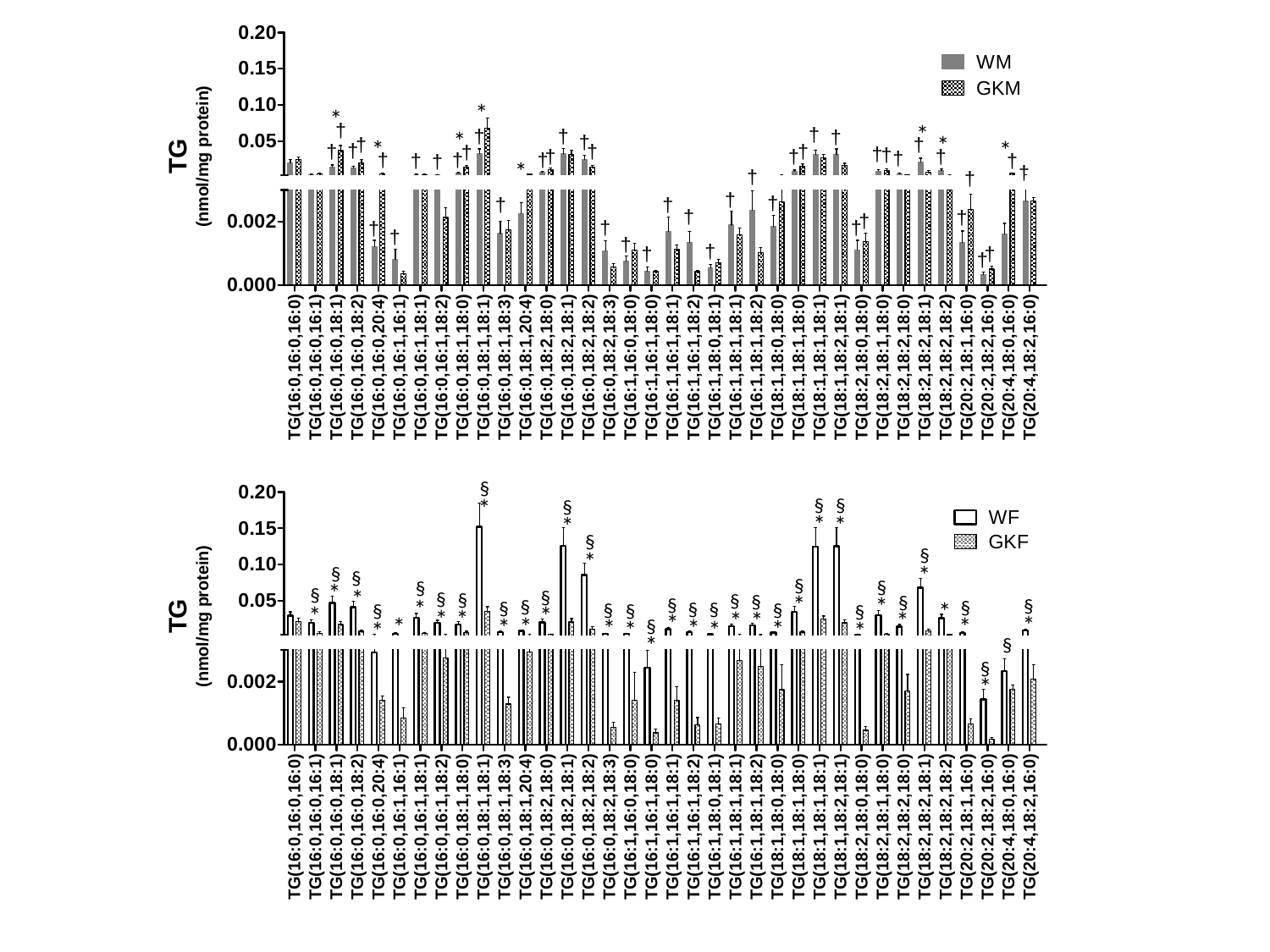

## Slide 2
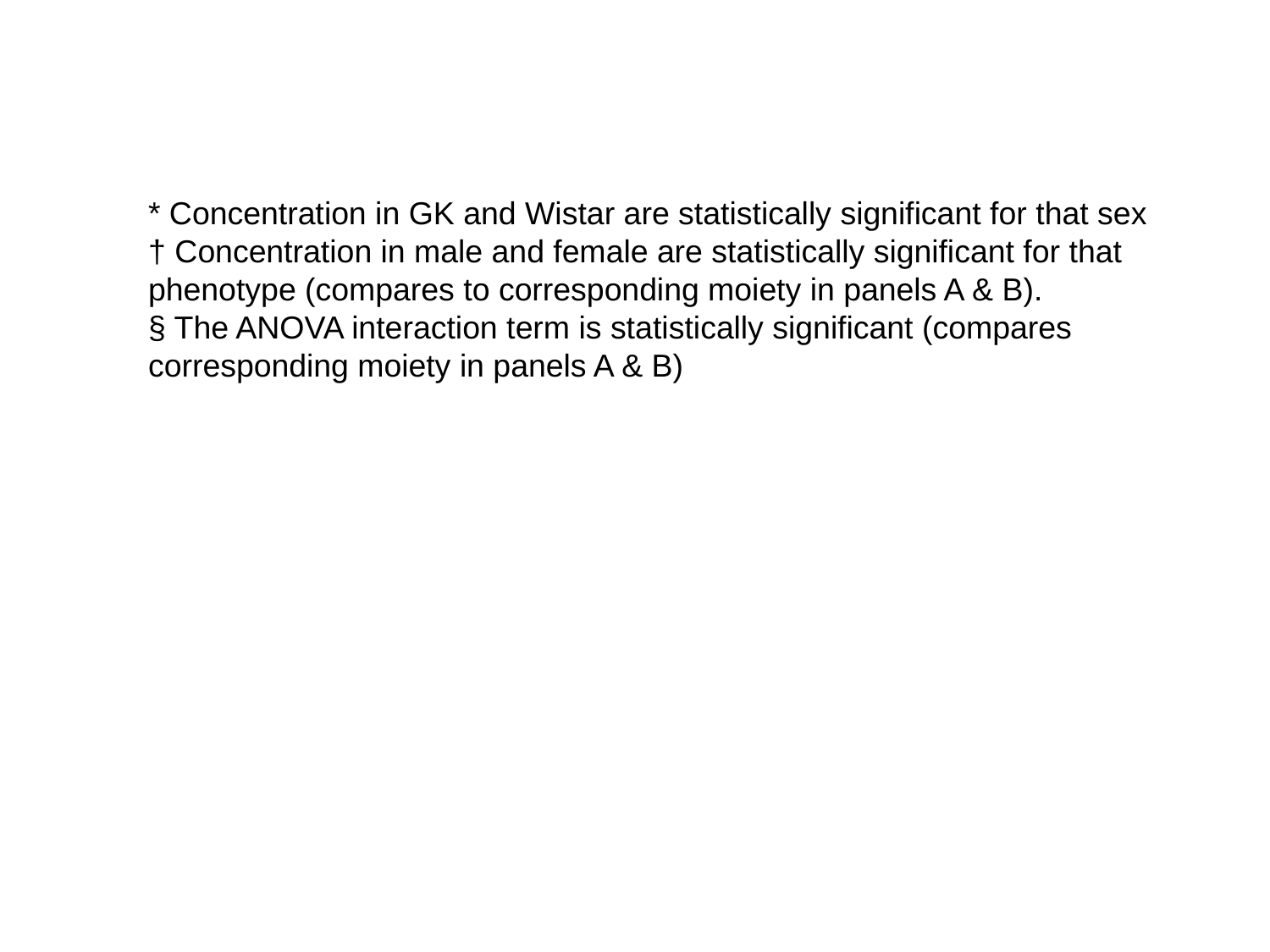

* Concentration in GK and Wistar are statistically significant for that sex
† Concentration in male and female are statistically significant for that phenotype (compares to corresponding moiety in panels A & B).
§ The ANOVA interaction term is statistically significant (compares corresponding moiety in panels A & B)
